# Function of Proneural Genes Ascl1 and Asense in Neurogenesis: How Similar Are They?

**DOI:** 10.3389/fcell.2022.838431

**Published:** 2022-02-18

**Authors:** Diogo S. Soares, Catarina C.F. Homem, Diogo S. Castro

**Affiliations:** ^1^ i3S Instituto de Investigação e Inovação em Saúde, IBMC Instituto de Biologia Molecular e Celular, Universidade do Porto, Porto, Portugal; ^2^ CEDOC, Nova Medical School, Faculdade de Ciências Médicas da Universidade Nova de Lisboa, Lisboa, Portugal

**Keywords:** Ascl1, asense, proneural bHLH transcription factors, neurogenesis, neural/stem progenitor cells

## Abstract

Proneural genes were initially identified in *Drosophila*, where pioneer work on these important regulators of neural development was performed, and from which the term proneural function was coined. Subsequently, their counterparts in vertebrates were identified, and their function in neural development extensively characterized. The function of proneural transcription factors in flies and vertebrates is, however, very distinct. In flies, proneural genes play an early role in neural induction, by endowing neural competence to ectodermal cells. In contrast, vertebrate proneural genes are expressed only after neural specification, in neural stem and progenitor cells, where they play key regulatory functions in quiescence, proliferation, and neuronal differentiation. An exception to this scenario is the *Drosophila* proneural gene *asense*, which has a late onset of expression in neural stem cells of the developing embryo and larvae, similar to its vertebrate counterparts. Although the role of Asense remains poorly investigated, its expression pattern is suggestive of functions more in line with those of vertebrate proneural genes. Here, we revise our current understanding of the multiple activities of Asense and of its closest vertebrate homologue Ascl1 in neural stem/progenitor cell biology, and discuss possible parallels between the two transcription factors in neurogenesis regulation.

## Introduction

In the developing nervous system, the specification and differentiation of neuronal cells relies on a class of proneural genes that encode basic-helix-loop-helix (bHLH) transcription factors (Huang et al., 2014). These evolutionary conserved genes were initially identified in *Drosophila melanogaster* in the 1980s given their ability to bestow neural identity onto naïve ectodermal cells, a property termed proneural function (Bertrand et al., 2002). *Drosophila* proneural genes include the four initially characterized members of the *achaete-scute* gene complex (AS-C)—*achaete* (*ac*), *scute* (*sc*), *lethal of scute* (*lsc*), and *asense* (*ase*)—as well as the later identified *atonal* (*ato*) and close-related genes *absent MD neurons and olfactory sensilla* (*amos*) and *cousin of atonal* (*cato*) (Garcia-Bellido, 1979; Villares and Cabrera, 1987; Ghysen and Dambly-Chaudière, 1988; Jarman et al., 1993a; Jarman et al., 1993b; Goulding et al., 2000a; Goulding et al., 2000b; Huang et al., 2000). Subsequently, two major classes of proneural genes were identified in the mouse: *achaete-scute* homologue *Ascl1*, and members of the *neurogenin* family, more related to *ato* (Johnson et al., 1990; Gradwohl et al., 1996; Ma et al., 1996; Fode et al., 1998). In contrast to flies, vertebrate proneural genes are expressed in progenitors already endowed with neural identity, suggesting they play later developmental functions. Accordingly, both gain- and loss-of-function studies in vertebrates showed proneural genes are both required and sufficient to induce neuronal differentiation, while also specifying neuronal subtype identities (Bertrand et al., 2002; Wilkinson et al., 2013). The classical definition of proneural function in *Drosophila* and vertebrates thus differs significantly: the former being associated with the acquisition of neural identify; the latter being associated with neuronal commitment.

A conserved feature of proneural genes across species is their ability to restrict their own expression in a non-cell-autonomous manner, by a process called lateral inhibition (Bertrand et al., 2002). Proneural factors induce the transcription of Notch ligands, ultimately activating the pathway in adjacent cells. Notch pathway activation results in expression of genes of the *enhancer of split* complex (*E(spl)-C*) or their vertebrate homologues *Hes/Her/Esr*, which in turn repress proneural gene expression (Bray, 2016). In *Drosophila*, proneural genes are detected initially at low levels in groups of ectodermal cells (proneural clusters). Lateral inhibition amplifies small differences in proneural gene expression, resulting in the segregation of discrete neural precursor cells. These are the Sensory Organ Precursor (SOP) cells of the Peripheral Nervous System (which will give rise to both external and internal sensory organs), and the Neuroblasts (NB)—the neural stem (NS) cells in the *Drosophila* Central Nervous System (Skeath and Carroll, 1991; Campuzano and Modolell, 1992). In vertebrates, lateral inhibition is transient, functioning to avert concurrent differentiation, and consequent depletion of the NS cell pool (Imayoshi and Kageyama, 2014). Activation of the Notch pathway is therefore a hallmark of proneural function across species. Nevertheless, the functions of *Drosophila* and vertebrate proneural factors in neural development have been perceived as highly divergent. Although this is the general rule, one gene of the *achaete-scute* complex—*ase*—does seem to tell a different story. Ase is not expressed in proneural clusters, but instead in neural precursor cells, including the NBs of the embryo and larvae (Gonzalez et al., 1989; Brand et al., 1993; Bowman et al., 2008; Álvarez and Díaz-Benjumea, 2018). This timing excludes it from the classical definition of a proneural gene in flies, suggesting a role more akin to its vertebrate counterparts. On the other hand, recent studies of vertebrate proneural factors revealed unexpected functions for these genes in NS and progenitor cells, prior to neuronal commitment. This is more evident in the case of Ascl1, the closest vertebrate homologue of Ase (Castro et al., 2011; Castro and Guillemot, 2011; Imayoshi et al., 2013; Andersen et al., 2014). Here, we discuss the similarities and differences between these two important proneural factors, in light of the current knowledge.

### Structural Comparison Between Ascl1 and Ase

**FIGURE 1:**
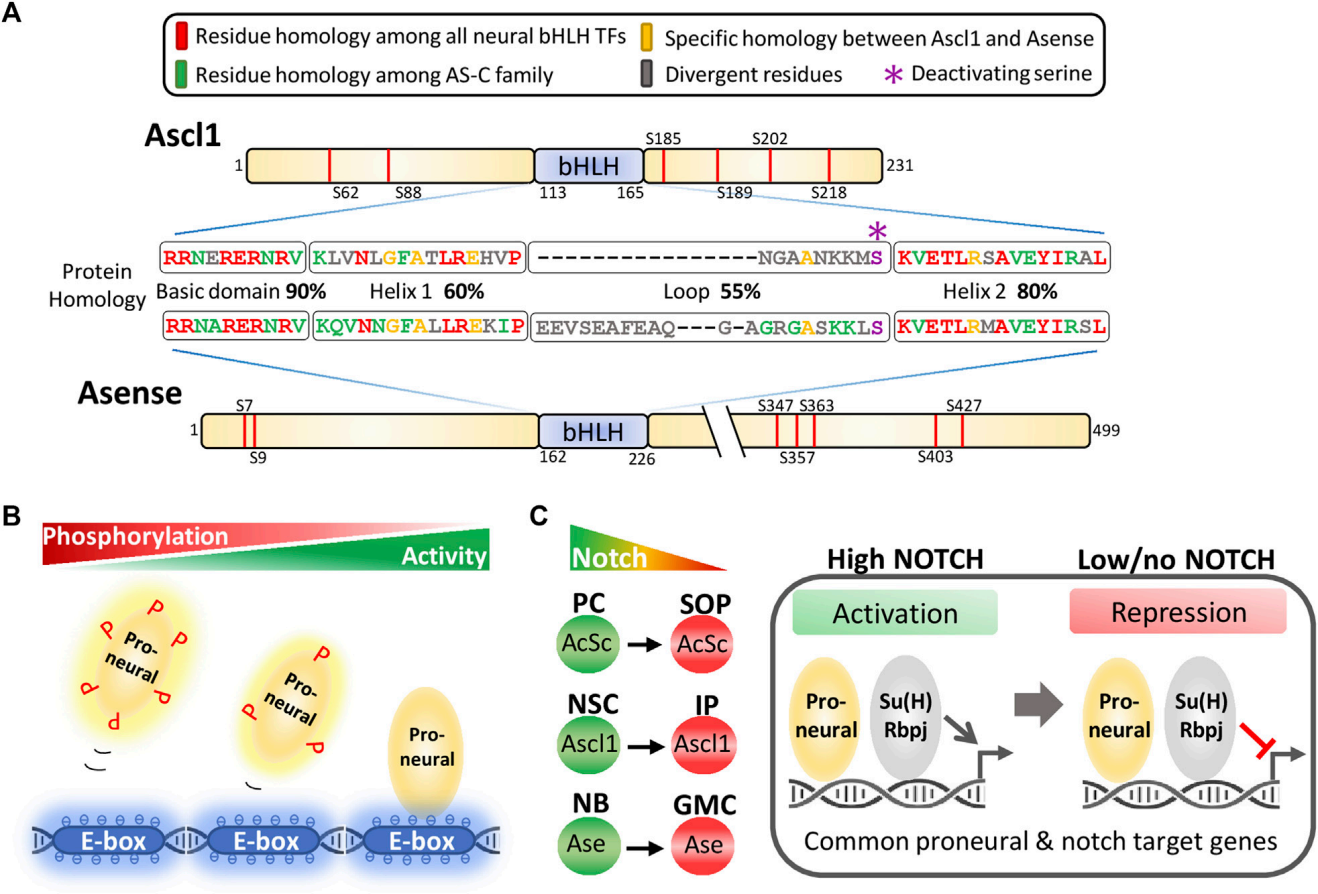
Structural comparison and mechanisms regulating proneural transcription factor activity. **(A)** Structural comparison between Asense and Ascl1 proteins. Schematic of both factors, showing protein sequence homology of their bHLH domains. Phosphorylation events at serine/proline sites (marked in red across N- and C- terminals) are shown for Ascl1 (described by the rheostat model) and Asense (predicted based on sequence). **(B)** Rheostat regulation of proneural factor activity. Multi-site phosphorylation modulates the electrostatic potential of proneural proteins, impacting their ability to interact with negatively charged chromatin. **(C)** Cell-autonomous cross-talk between proneural and Notch pathways, as originally described in the developing peripheral nervous system of Drosophila. Left: Proneural proteins are expressed in cells with distinct Notch signalling levels (i.e., cells with distinct levels of notch receptor activation). Right: In cells with high Notch levels, the downstream effectors of Notch pathway Su(H) and Rbpj function as transcriptional activators, activating the expression of common Notch/proneural target genes in synergy with Ascl1 (right). When Notch signalling is low, Su(H)/Rbpj represses common targets in Ascl1 expressing cells. PC, proneural cluster; SOP, sensory organ precursor; NSC, neural stem cell; IP, intermediate precursor; NB, neuroblast; GMC, ganglion mother cell.

As class II bHLH transcription factors, proneural proteins bind DNA in a heterodimer complex with class I bHLH transcription factors (also designated by *E-proteins*) (Bertrand et al., 2002). While in vertebrates there are five E-proteins (e.g., E47, E12) product of three genes, in *Drosophila* the sole E-protein is encoded by *daughterless* (*da*) (Ledent and Vervoort, 2001). Dimerization is mediated by the HLH domain of each partner, while the basic domain is required for sequence-specific binding to consensus sequences (E-boxes). All members of the *Drosophila* AS-C complex share total homology within their basic domains, and 90% homology with the basic domains of both mouse and human Ascl1 (Figure 1A). In line with this high conservation, similar consensus E-boxes were determined for Ascl1, and Ase (Southall and Brand, 2009; Raposo et al., 2015).

To compare the developmental functions of Ascl1 and Ase in each native context, it is important to understand to which extent Ascl1 and Ase proteins are functionally equivalent. Relevant studies have shown the *Drosophila* gene *ato* can completely rescue the complex developmental phenotype of mouse embryos null to its homologue *Atoh1*, (Hassan and Bellen, 2000), whereas Ase can replace other AS-C genes if expressed in proneural clusters (Brand et al., 1993). In both cases there is little or no conservation outside the bHLH domain, suggesting that proneural specificity is to large extent determined by this protein region. Thus, the high conservation between Ascl1 and Ase bHLH domains suggest the two factors may be functionally interchangeable to a large extent. Nevertheless, highly divergent N- and C-terminal domains may allow for differences in how their activity is fine-tuned, for example by post-translational modifications (PTMs) (Figure 1A).

### Multiple Ascl1 Functions Along the Neuronal Lineage

**FIGURE 2:**
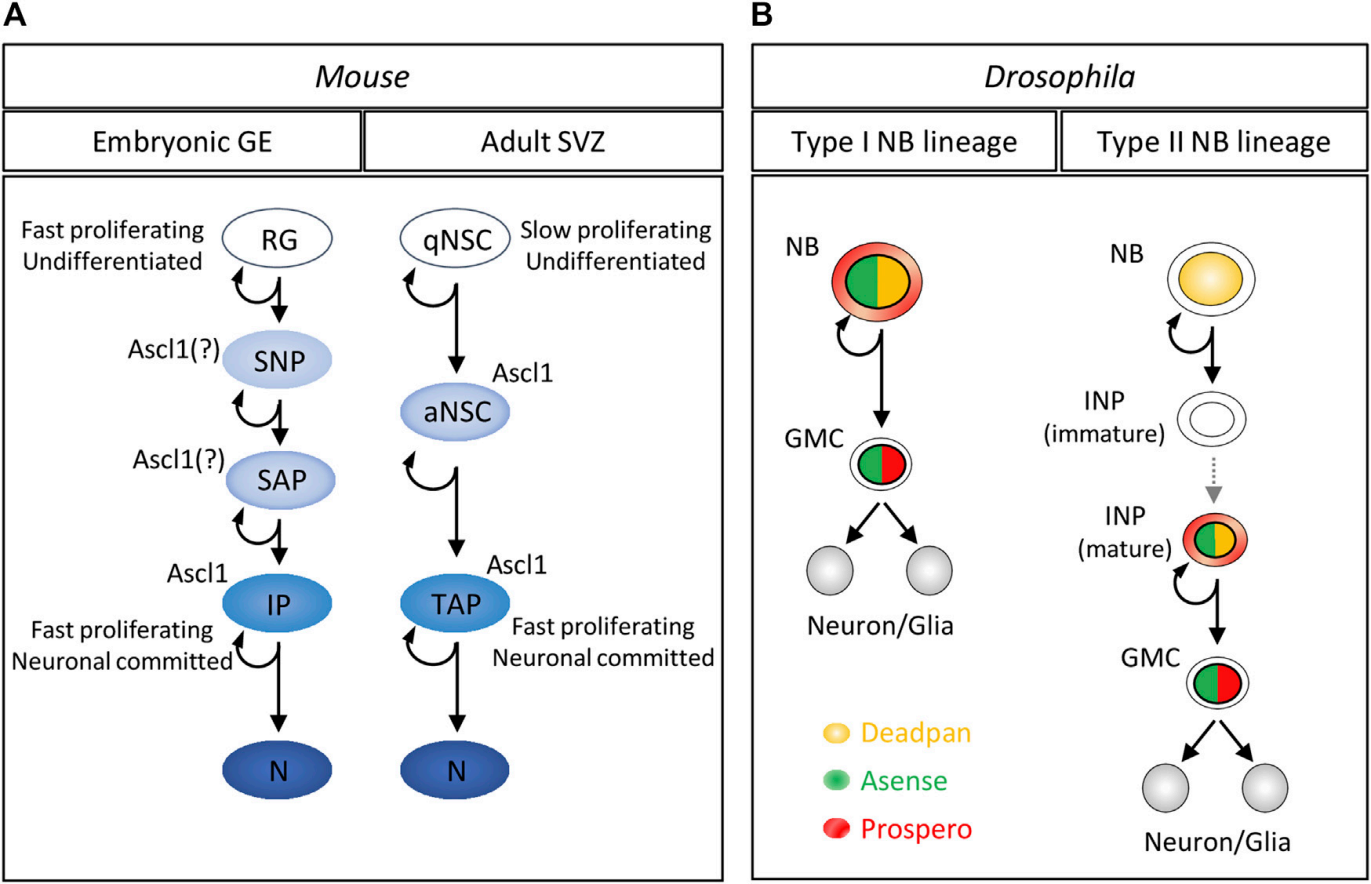
Expression of Ascl1 and Ase in neurogenic lineages of vertebrates and flies **(A)** Schematic diagram of an embryonic (lateral ganglionic eminence) and an adult (lateral ventricle) neurogenic lineage in mouse brain. In the embryonic lineage, Ascl1 expression is excluded from Radial Glia neural stem cells, starting in more restricted progeny. In the adult SVZ, Ascl1 expression starts in activated NS cells as they exit quiescence, being maintained in fast proliferating, neuronal committed progenitors. Blue color gradient indicates progression along the neuronal lineage GE, ganglionic eminence; SVZ, sub-ventricular zone; RG, Radial glia; SNP, short neural precursors; SAP, sup-apical progenitor; IP, intermediate progenitor; N, neuron. qNSC, quiescent neural stem cell; aNSC, activated neural stem cell; TAP, transiently amplifying progenitor. **(B)** Schematic diagram of the division patterns of type I and type II neuroblast lineages in the central brain of *Drosophila* larvae. Ase is expressed in Type I NBs that divide asymmetrically to self-renew and produce a smaller GMC, which maintains Ase expression. By contrast, Type II neuroblasts lack Ase expression. In this lineage, Ase expression starts in INPs as they mature, and is subsequently terminated in GMCs upon nuclear translocation of Pros (which triggers an irreversible decision towards differentiation). Colors represent cytoplasmic or nuclear expression of each transcription factor—Dpn (yellow); Ase (green); Pros (red). NB, neuroblast; GMC, ganglion mother cell; N, neuron; INP, intermediate neural progenitor.

Ascl1 expression is spatially restricted to diverse progenitor domains along the rostro-caudal axis of the developing brain and spinal cord (Vasconcelos and Castro, 2014). In embryonic neurogenesis, the role of Ascl1 has been best scrutinised in the ventral domain of the telencephalon, the most rostral division of the embryonic brain (Casarosa et al., 1999). Live-cell imaging of the germinal layers at the lateral ganglionic eminence (LGE) in the ventral telencephalon described a complex lineage, with Radial Glia (RG) NS cells in the Ventricular Zone (VZ) at the top of a hierarchy that includes apically-dividing short neural precursors (SNPs), sub-apically dividing progenitors (SAPs), and intermediate progenitors (IPs) that divide in the Sub Ventricular Zone (SVZ) (Pilz et al., 2013; García and Harwell, 2017). In the LGE, Ascl1 is excluded from (Gsx2 expressing) RG cells (Roychoudhury et al., 2020), starting to be expressed in apically dividing progenitors, most likely SNPs (Soares et al., 2021) (Figure 2A).

Gain-of-function studies support a role for Ascl1 in driving neuronal differentiation and specification in the embryo (Farah et al., 2000; Parras et al., 2002; Nakada et al., 2004). Furthermore, Ascl1 overexpression in chick spinal cord results in cell-cycle exit of progenitors, migration and subsequent expression of pan-neuronal and neuronal subtype-specific markers (Nakada et al., 2004). Conversely, *Ascl1* ablation in mouse results in decreased neuronal progeny in the embryo, as observed in the telencephalon, characterized by reduced basal ganglia neurons, and specific interneuron populations (Casarosa et al., 1999). Somehow at odds with the classical view of mammalian proneural gene function, additional studies revealed an unexpected role for Ascl1 in promoting proliferation prior to differentiation. Accordingly, Ascl1 null embryos exhibit reduced proliferation in the VZ and SVZ, with acute Ascl1 knock-down in the ventral telencephalon resulting in progenitors prematurely withdrawing from cell-cycle and differentiating (Casarosa et al., 1999; Castro and Guillemot, 2011). Moreover, decreased proliferation is observed in cultures of embryonic NS cells upon acute knock-down of Ascl1, or when these cultures are established from constitutive Ascl1 null embryos (Castro and Guillemot, 2011; Imayoshi et al., 2013).

More recently, conditional ablation of *Ascl1* provided important insights into its role in both neurogenic niches of the adult rodent brain—the SVZ of the lateral ventricle, and the Sub Granular Zone of the Dentate Gyrus in the Hippocampus (Andersen et al., 2014; Urbán et al., 2016). While embryonic NS cells are highly proliferative, most NS cells in the adult brain are found in a quiescent state, and devoid of Ascl1 expression (Figure 2A). Strikingly, exit from quiescence is entirely dependent on Ascl1, with conditional ablation of *Ascl1* in the adult brain halting neurogenesis in both neurogenic niches (Andersen et al., 2014). In adult lineages, Ascl1 expression starts in activated NS cells, as these exit quiescence, being maintained in more differentiated progeny. In the SVZ of the lateral ventricle, Ascl1 protein is detected in rapidly proliferating transit amplifying progenitors (TAPs) and in a small fraction of neuroblasts, migrating towards the olfactory bulb (Parras et al., 2004). This suggests the dual role of Ascl1 (promoting sequentially proliferation and differentiation) is maintained in the adult, where its pro-proliferative function is less redundant with other pathways as compared to embryonic stages (Casarosa et al., 1999; Andersen et al., 2014).

Important insights into how Ascl1 coordinates neurogenesis were obtained upon the genome-wide characterization of its transcriptional targets in embryonic regions such as the ventral telencephalon and dorsal spinal cord (Castro and Guillemot, 2011; Borromeo et al., 2014). In agreement with its pro-proliferative role, these targets include transcription factors promoting cell-cycle progression (e.g. *E2F1*, *FoxM1*), and cell-cycle regulators (e.g. *Cdk1/2*, *Ccnd2*) (Castro and Guillemot, 2011). Ascl1 targets include also genes with a predicted role in neuronal differentiation, migration, axon guidance or synapse formation, and indicating Ascl1 exerts a direct control over multiple components of the neurogenic program. In line with this complex model, Ascl1 targets in the ventral telencephalon have diverse onsets of expression: in undifferentiated progenitors (predominantly VZ), in differentiating progenitors (predominantly SVZ), or even later in new-born neurons (mantle zone). Overall, the master regulatory function of Ascl1 in neurogenesis is reinforced by the extensive use of Ascl1 in reprogramming somatic cells into induced neurons, attributed to its ability to bind nucleosomal DNA, and promote chromatin accessibility (i.e. pioneer transcription factor activity) (Vierbuchen et al., 2010; Wapinski et al., 2013; Raposo et al., 2015).

### Ase Expression in NBs and Their Progeny

In the *Drosophila* embryo, Ase protein starts being expressed in cells segregating from the neuroectodermal epithelium, being absent from surrounding proneural cluster cells (Brand et al., 1993; Cubas et al., 1991; Gonzalez et al., 1989; Jarman et al., 1993a). These Ase expressing cells will originate most embryonic NBs, wherefrom neuronal cells are generated throughout the entire neurogenic period, from embryo to larval, and pupal stages. Ase expression has been best characterized in NB lineages in larvae, where two main types of NBs (type I and type II) are distinguished by different lineage trees (Figure 2B) (Bowman et al., 2008). Type I NBs, in both the central brain and ventral nerve cord, divide asymmetrically to self-renew, and produce a smaller Ganglion Mother Cell (GMC) that subsequently divides terminally into two neurons or glia. These NBs are characterized by the expression of Ase, along with nuclear Deadpan (Dpn), and cytoplasmic Prospero (Pros). The transition from a NB to a more fate restricted GMC is driven by increased Pros activity (resulting from its nuclear translocation), and occurs concomitantly with degradation of Dpn (Choksi et al., 2006; Doe et al., 1991; Li and Vaessin, 2000). In the type I lineage, Ase expression can be detected both at transcript and protein level in GMCs, before being repressed by Prospero (Bowman et al., 2008; Brand et al., 1993). By contrast to Type I NBs, a smaller number of Type II NBs in the central brain are characterized by Dpn expression, but lack both Ase and Pros. These NBs undergo multiple rounds of asymmetric divisions to self-renew and produce Intermediate Neural Precursors (INPs) (Álvarez and Díaz-Benjumea, 2018; Bayraktar et al., 2010; Boone and Doe, 2008; Bowman et al., 2008). INPs only start expressing Dpn, Ase and Pros after a period of maturation, proceeding to divide asymmetrically to self-renew and generate GMCs (Figure 2B). In the type II lineage, Ase expression in GMCs is again terminated after nuclear translocation of Pros, which directly represses Ase and other NB genes (e.g., Dpn, Miranda, and Inscuteable) to initiate differentiation (Choksi et al., 2006; Southall et al., 2008). Thus, mature INPs share many similarities with Type I NBs, including mode of division (asymmetric division, although limited in number for INPs) and expression of regulators such as Ase.

### Evidence for a Dual Function of Ase

Despite its expression pattern, Ase loss-of-function results in very mild phenotypes, which are nevertheless in line with a late developmental role, including morphological defects of the row of stout bristles in the wing margin, and or misrouting of axons in the optic lobe (Jarman et al., 1993a; Brand et al., 1993). It is possible, however, that the Ase null phenotype is masked by compensatory expression of other AC-S complex genes. DamID mapping of Ase binding sites in the embryo, combined with transcriptional profiling upon Ase knock-down in either NBs or GMCs, and revealed Ase target genes in each cell type (Southall and Brand, 2009). Genes activated by Ase in NBs, exemplified by *miranda* (required for asymmetric cell division), or *grainy head* (encoding positional identity), support an important role for Ase in the regulation of NB maintenance and self-renewal. Binding of Ase to differentiation genes, which are upregulated in Ase null NBs and GMCs, suggests an unexpected role for Ase in counteracting NB differentiation. However, this interpretation entails Ase repressing gene transcription, an activity not expected from proneural transcription factors. Dichotomously, this experimental approach also identified differentiation genes activated by Ase in NBs and GMCs. These display various biological functions, as exemplified by *dacapo* (cell-cycle exit), *commissureless* (axon guidance) or *hikaru genki* (synaptogenesis). The finding that Ase directly activates Pros is another indication of its role in differentiation, even though the activity of Pros is mostly regulated by its cellular localization (Hirata et al., 1995; Ikeshima-Kataoka et al., 1997). Moreover, regulation of some of the targets described above suggest a role for Ase in neuronal maturation. A specific role for Ase in neuronal fate would be in line with the prevalent view that glia specification and differentiation requires suppression of Ase (Badenhorst, 2001; Jones, 2005). However, some embryonic GMCs divide asymmetrically to produce one neuron and one glial cell. Thus, at least in some cases cell fate (i.e., neuronal *vs.* glia) cannot be solely determined by the presence/absence of Ase in the GMC.

Additional evidence supports a pro-differentiation function of Ase. Ectopic expression of Ase in Type II NBs restricts lineage expansion (resulting in Type I-like NBs), once again via upregulation of Pros (Bowman et al., 2008; Bayraktar et al., 2010). In optic lobe NBs, gain and loss-of-function of Ase results in decreased or increased mitotic activity respectively, via differential expression of its target *dacapo* (Wallace et al., 2000). Although a dual role for Ase in NB maintenance and differentiation has been better defined in the embryo, its sequential expression in both undifferentiated NBs, and in more differentiated GMCs (Type I lineage), and to some extent in mature INPs and GMCs (Type II lineage), suggests dual activity may also be a property of Ase in the larva (Bowman et al., 2008).

At the end of embryogenesis, *Drosophila* NS cells enter a period of dormancy termed *quiescence*, ceasing to generate GMCs*.* Proliferation is resumed during larval stages, stimulated in response to feeding upon larval hatching (Britton and Edgar, 1998; Sousa-Nunes et al., 2011; Homem and Knoblich, 2012; Homem et al., 2015). Quiescent NBs are characterized by the expression of Dpn, but not Ase (Lai and Doe, 2014). This quiescent state is induced by a transient pulse of low-level expression of nuclear Pros, which represses a transcriptional program that includes most NB markers (e.g., Ase, Miranda) except Dpn, and cell-cycle genes (e.g., *cyclin E*) (Lai and Doe, 2014). However, while Ase is repressed when cells become quiescent, maintaining its expression does not affect the timing of this cell-state transition (Lai and Doe, 2014). Interestingly, the absence of Ase expression in quiescent NBs is analogous to the lack of Ascl1 expression in quiescent adult NS cells. Whether Ase plays a role similar to Ascl1 in promoting exit from quiescence, remains undetermined.

### A Quantitative Model of Ascl1 Function

Mechanistically, Ascl1 enhances the proliferation of NS cells when it oscillates, and neuronal differentiation when its expression becomes sustained (Imayoshi et al., 2013). These two modes of Ascl1 expression (oscillatory versus sustained) are part of a revised view of the lateral inhibition model in vertebrates, which has at its core the ability of Hes1 to function as an intrinsic oscillator (Kageyama et al., 2008; Pierfelice et al., 2011). While in proliferating NS cells Hes1 oscillatory behavior induces Ascl1 oscillations in antiphase, downregulation of Hes1 at onset of differentiation results in sustained expression of Ascl1. How does oscillatory versus sustained expression of Ascl1 results in sequential proliferation and differentiation along the neuronal lineage? The current view suggests a quantitative model, whereby low Ascl1 activity promotes (and is compatible with) progenitor cell proliferation, whereas an increase in Ascl1 activity results in cell-cycle-exit and differentiation (Vasconcelos and Castro, 2014). Since these two Ascl1 functions are associated with differential gene activation, it is reasonable to assume that distinct target genes respond differently to Ascl1 activity levels. The chromatin landscape is a likely determinant, as suggested by higher chromatin accessibility at Ascl1 bound enhancers of progenitor genes vs. differentiation genes, in proliferating neural NS cells (Raposo et al., 2015).

Considering the above model, future studies should clarify whether quantitative differences in Ase transcriptional activity determine distinct cellular functions. This will require a better characterization of Ase protein levels across different cell contexts, and investigating if any putative differences impact cell fate decisions. It is unlikely however, that oscillations resembling the ones described for Ascl1 regulate Ase activity, given the extremely short cell-cycle characteristic of *Drosophila* NBs (approximately 1 hour) (Bowman et al., 2008).

### Regulation of Proneural Factor Activity by Multisite Phosphorylation

Besides oscillatory expression, PTMs may provide another mechanism to down-regulate the transcriptional activity of Ascl1 in proliferating cells (Guillemot and Hassan, 2017). Accordingly, two studies proposed phosphorylation of Ascl1 at six serine-proline (SP) sites (outside its DNA binding domain), to control the balance between Ascl1 proliferating/differentiating activities. One study found phosphorylation of these SP sites could be promoted by CDK1/2, decreasing the differentiation activity of Ascl1 in a neurogenesis assay in Xenopus embryos, and or in a neuronal reprogramming protocol in mammalian cells (Ali et al., 2014) (Figure 1A). The second study showed phosphorylation of the same six residues can occur downstream of RAS/ERK signaling, resulting in a proliferative/gliogenic phenotype at the expense of neuronal differentiation (Li et al., 2014). Taken together, evidence suggests that some level of constitutive phosphorylation of Ascl1 in cycling cells occurs in combination with more dynamic phosphorylation downstream regional and developmental specific pathways, decreasing its neurogenic activity.

Multisite phosphorylation has been shown to regulate the activity of other vertebrate proneural factors, via a mechanism that relies on the total number of negatively charged phosphoresidues (not their specific location) (Ali et al., 2011; Hindley et al., 2012; Azzarelli et al., 2017). This suggests a rheostat-like mechanism based on gradual changes of electrostatic potential by multisite phosphorylation, regulating the interaction of proneural proteins with negatively charged chromatin (Figure 1B). Future work should address whether different phospho-status of Ascl1 impact its interaction with specific chromatin states, helping to determine target gene selection.

Interestingly, phosphorylation of a conserved serine/threonine residue in the bHLH domain works as a binary switch across *Drosophila* and vertebrate proneural proteins (governing the duration of their activity), providing a precedent for an evolutionarily conserved mechanism controlling proneural function based on PTMs (Figure 1A) (Quan et al., 2016). However, to which extent the rheostat model could be extended to the fly, is not known. Multisite phosphorylation of SP sites takes place at the highly divergent N- and C-terminal domains of proneural proteins, as it is the case with Ascl1 (Ali et al., 2014). In Ase, a total of 7 SP sites are found similarly distributed outside its bHLH domain, along the N- and C-terminus, suggesting multisite phosphorylation (namely by CDKs) may also regulate Ase function (Figure 1A).

### A Cell-Autonomous Cross-Talk With the Notch Pathway

Previous studies in the developing peripheral nervous system of *Drosophila* revealed how a cell-autonomous crosstalk with the Notch pathway provides context dependency to proneural AS-C proteins (Figure 1C) (Castro et al., 2005). SOP selection is associated with increased expression of proneural proteins, and concomitant downregulation of Notch signalling. During this process, several genes of the E (spl)-C (e.g., *E*(*spl*)*mα*, *E*(*spl*)*m8*) are simultaneously controlled by both AS-C proteins and Suppressor of Hairless [Su(H)] (the downstream Notch effector) (Nellesen et al., 1999; Cave et al., 2011). Su(H) functions as a transcriptional switch, promoting activation or repression, depending on Notch signalling status. As a result, co-recruitment of proneural and Su(H) transcription factors to regulatory enhancers of proneural targets, results in: 1) synergetic activation of transcription between proneural and Notch pathways in cells with high Notch signalling (proneural clusters), and 2) default repression by Su(H) in the absence of Notch signalling (SOPs). Thus, such cross-talk allows for the down-regulation of proneural target genes, during a developmental step associated with increased proneural activity. This paradigm can be reproduced in transcriptional assays using Ascl1, and may thus be conserved in vertebrate neurogenesis where lineage progression is also associated with decreased Notch signalling (Cave et al., 2005). In support of this possibility, the consensus binding sequence for Rbpj (homologous of Su(H)) was found enriched specifically in the vicinity of Ascl1 binding sites at proliferation genes (Castro and Guillemot, 2011). Interestingly, the characterization of Notch targets in larval NBs revealed a strong overlap with previously characterized Ase program (Zacharioudaki et al., 2016). This suggests the same model may also be applicable to Ase, which is also expressed in cell types with distinct Notch signalling levels. The use of a Notch reporter in the Type I lineage revealed that undifferentiated NBs and more differentiated GMCs (both of which express Ase) are characterized by high and low Notch signalling, respectively (Almeida and Bray, 2005). A similar situation is found in the Type II lineage, where sequential expression of Ase in mature INPs and GMCs occurs with concomitant decrease of Notch pathway activity (Almeida and Bray, 2005).

## Conclusion

The pivotal role of Ascl1 in vertebrate neurogenesis has been extensively characterized in recent years. In contrast, comparatively little is known on the biological function of its fly counterpart Ase, a widely-used marker for Type I NBs. Nevertheless, some similarities and differences have started to emerge. Neither Ase or Ascl1 are required for the early acquisition of NS cell identity, playing instead later regulatory roles associated with their expression in NS cells, and some of their progeny. Interestingly, both transcription factors have been shown to coordinate different components of the neurogenesis program by performing dual, and albeit different, sequential functions along the lineage. A proliferative function of Ascl1 in NS cells (prior to its differentiation role) has been shown in both embryonic and adult stages. In contrast, no evidence of such function has been shown for Ase, which nevertheless regulates positional identity and self-renewal of NS cells. In parallel with the well-established role of Ascl1 in neuronal commitment and differentiation, some observations suggest Ase can drive genes involved in cell-cycle exit, and neuronal maturation in NS cell progeny. However, to which extent these functions are conserved in multiple neuronal lineages, is an important question that remains unresolved. At the molecular level, future work should also clarify whether mechanisms regulating Ascl1 activity are applicable to Ase, and most notably regulation by multi-site phosphorylation. *Drosophila melanogaster* has served as a tremendously valuable model to uncover developmental mechanisms conserved in vertebrates. Obtaining a clearer understanding of the role of Ase may elucidate on further unknown mechanisms by which Ascl1 regulates neurogenesis.
